# An Interstitial Deletion at 10q26.2q26.3

**DOI:** 10.1155/2014/505832

**Published:** 2014-02-06

**Authors:** Ivan Y. Iourov, Svetlana G. Vorsanova, Oxana S. Kurinnaia, Yuri B. Yurov

**Affiliations:** ^1^Mental Health Research Center, Russian Academy of Medical Sciences, Zagorodnoe Shosse 2, Moscow 117152, Russia; ^2^Institute of Pediatrics and Children Surgery, Ministry of Health of Russian Federation, Moscow 125412, Russia; ^3^Department of Medical Genetics, Russian Medical Academy of Postgraduate Education, Moscow 123995, Russia; ^4^Moscow City University of Psychology and Education, Moscow 127051, Russia

## Abstract

We present a case of an interstitial subtelomeric 10q26 deletion in a male child with moderate developmental delay and minor dysmorphic features. Using array comparative genomic hybridization (CGH) and fluorescence *in situ* hybridization (FISH), we have detected an interstitial deletion at 10q26.2q26.3 encompassing a 5.8 Mb region and spanning 24 genes. Interestingly, losses of this chromosome 10 region have not been previously associated with a phenotype outcome. According to an *in silico* evaluation, we have suggested that *PPP2R2D* and *BNIP3* losses are likely a cause of developmental delay in the index patient. Our data allow to speculating that haploinsufficiency of these two genes in 10q26.3, which is usually ignored in the context of chromosome 10q deletions, has a phenotypic effect.

## 1. Introduction

Although subtelomeric chromosomal rearrangements are common in children with intellectual disability, developmental delays and/or dysmorphic features [1–3], deletions affecting subtelomere of chromosome 10 long arm, are rare [4]. This probably explains the small amount of such cases addressed by array comparative genomic hybridization (CGH) [4, 5] in contrast to the number of reports on phenotypic manifestations of chromosome 10q25q26/10q26qter loss analyzed using molecular cytogenetic techniques with a lower resolution [6–8]. Here, we report a case of an interstitial 10q26 deletion in a male child presenting with a phenotypic outcome atypical for subtelomeric deletions at 10qter detected by array CGH and confirmed by fluorescence *in situ* hybridization (FISH).

## 2. Case Presentation and Methods

### 2.1. Clinical Description

A 28-month-old male child with moderate developmental delay and minor dysmorphic features has been referred to (molecular) cytogenetic analysis. The patient was born at 37 weeks of gestation to a 20-year-old mother and 21-year-old father by spontaneous vaginal delivery with a birth weight of 3.16 kg (~25th percentile) and length of 52.6 cm (~75th percentile). Pregnancy history was unremarkable. At 6 months of age, he was able to roll over and sit with support. Physical examination at the age of 2 years and 4 months showed dysmorphic features (flat feet with cutaneous syndactyly of the second and third toes; high forehead and prominent auricles) without any other remarkable congenital malformations and dysmorphic features. A moderate developmental delay was noticed.

### 2.2. Cytogenetic Analysis

GTG-banding was done according to standard procedures analyzing 30 metaphase plates at a resolution of 550 bands. Karyotype abnormalities were not detected.

### 2.3. Array CGH

**Figure 1:**
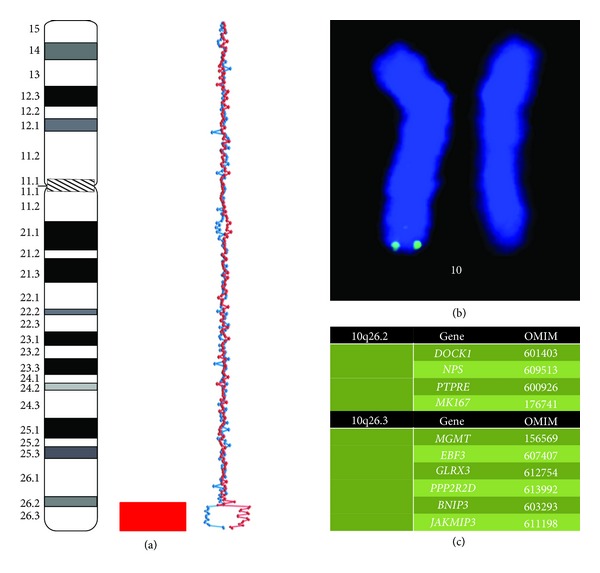
Molecular cytogenetic (array CGH) findings in the index case: (a) array CGH demonstrating arr 10q26.2q26.3(128,190,760–133,998,503) × 1 (two alternative arrays Cy3/Cy5 (red line) and Cy5/Cy3 (blue line) are plotted on the graph); (b) FISH confirmation of subtelomeric 10q deletion; (c) OMIM genes (http://www.omim.org) located at the deleted chromosome 10 region.

Array CGH was performed using the customized human genomic microarrays (slightly modified Constitutional Chip 4.0) BAC/PAC clones (Human BAC Array-System, Perkin Elmer, USA) achieving a resolution of 0.3 Mb. Technical performance of array CGH (DNA labeling, hybridization, detection, and data analysis) was done according to previously described protocols [9, 10] and to manufacturers' instructions. An interstitial deletion at 10q26.2q26.3 spanning 128,190,760–133,998,503 (confirmed by 14 interchangeable BAC probes (two reverse assays): RP11-16P8, RP11-32H11, RP11-21M8, RP11-42K2, RP11-88B12, RP11-48A2, RP11-168C9, RP11-90B19, RP11-90O13, RP11-113P9, RP11-462G8, RP11-408L20, RP11-142I8, and RP11-140A10) was detected (Figure 1(a)). The deletion size was estimated as 5.8 Mb.

### 2.4. FISH

FISH was performed as described elsewhere [11–13] with a DNA probe located at chromosome 10q26 [12]. Subtelomeric deletion of chromosome 10q was confirmed (Figure 1(b)).

### 2.5. *In Silico* Evaluation of the Deleted Chromosomal Region

Bioinformatics analysis of the deletion was made as proposed in our previous studies [9, 10, 13]. The deletion resulted in the loss of 24 genes among which 10 genes are listed in OMIM (http://www.omim.org/) (Figure 1(c)). Using a set of genomic, epigenetic and proteomic databases (for details see [9, 10, 13]), we have evaluated the pathogenic value of 10q26 genes' haploinsufficiency caused by the deletion. We concluded that *PPP2R2D* and *BNIP3* losses are likely to be the cause of intellectual disability in the index patient.

## 3. Discussion

Subtelomeric chromosome 10q deletions appear to cause a specific phenotype [4–8]. However, wide clinical heterogeneity naturally associated with variability in deletion size has been reported [4, 14]. Interestingly, phenotypic outcomes of all the deletions at 10q26 addressed by array CGH have not been ever associated with haploinsufficiency of *PPP2R2D* and *BNIP3* genes. Molecularly, the detected deletion is similar to a genomic variation, which had resulted from a complex distal 10q rearrangement in a girl with mild intellectual disability reported by Sarri et al. [15]. Unfortunately, the complex chromosome rearrangement has involved other genomic loci impeding correct phenotype correlations. Additionally, Courtens et al. [16] have described subtelomeric 10q deletion spanning the same 10q26.2 genomic loci associated with phenotypically different outcomes. Moreover, bioinformatics analysis has shown that the phenotype is likely to result from simultaneous loss of *PPP2R2D* and *BNIP3*. Therefore, we have concluded these two genes to be implicated in the phenotypic outcome of interstitial deletions affecting 10q26.2q26.3. Finally, the present case demonstrates that chromosomal loci ignored in the context of genomic disorders can contribute to the phenotype or cause mild subtypes of the disease.
